# Effect of Proximity, Burden, and Position on the Power Quality Accuracy Performance of Rogowski Coils

**DOI:** 10.3390/s22010397

**Published:** 2022-01-05

**Authors:** Alessandro Mingotti, Federica Costa, Lorenzo Peretto, Roberto Tinarelli

**Affiliations:** Department of Electrical, Electronic and Information Engineering, Guglielmo Marconi Alma Mater Studiorum, University of Bologna, Viale del Risorgimento 2, 40136 Bologna, Italy; federica.costa13@unibo.it (F.C.); lorenzo.peretto@unibo.it (L.P.); roberto.tinarelli3@unibo.it (R.T.)

**Keywords:** low-power instrument transformer, Rogowski coil, position, burden, proximity, accuracy, harmonic, power quality

## Abstract

Power quality evaluation is the process of assessing the actual power network parameters with respect to the ideal conditions. However, several new assets and devices among the grid include mining the voltage and current quality. For example, the power converters needed for renewable energy sources’ connection to the grid, electric vehicles, etc., are some of the main sources of disturbances that inject high-frequency components into the grid. Consequently, instrument transformers (ITs) should be capable of measuring distorted currents and voltages with the same level of accuracy guaranteed for the ideal frequency (50–60 Hz). This is not a simple task if one considers that several other influence quantities endlessly act on the ITs. To this purpose, considering the lack of a standard, this work presents a measurement setup and specific tests for testing a commonly used type of low-power current transformer, the Rogowski coil (RC). In particular, the accuracy performance (ratio error and phase displacement) of the RCs was evaluated when measuring distorted signals while other influence quantities affected the RCs. Such quantities included positioning, burden, and magnetic field. The results indicate which quantities (or combination of them) have the greatest effect on the RC’s accuracy performance.

## 1. Introduction

The world of instrument transformers (ITs) is constantly evolving and adapting to the innovations that the power network is experiencing. Since the end of the 20th century, after the introduction of decentralized renewable energy sources (RES), the grid has observed its first major changes. This “small” step changed the legacy structure of the grid, introducing a new concept of bidirectional power flow [1,2]. The consequences for the system operators (SOs) include increasing difficulty in the managing and controlling of the network.

In recent years, the situation has further worsened, considering the advent and availability of smart technologies such as smart meters, intelligent electronic devices (IEDs), electric vehicles (EVs), and the spread of RES at the low-voltage (LV) customer level.

For example, smart meters and IEDs enabled a variety of operations to be performed on the current and voltage measurements. Among them, power, energy, and PQ measurements are among the most important. Nevertheless, some drawbacks arose in parallel with the benefits: (i) in the distribution network (DNs) the deployment of IEDs involves thousands or hundreds of thousands of nodes to be instrumentalized [3,4]; (ii) the amount of data resulting from the devices is critical either from a security and privacy perspective or for their analysis [5,6].

As for the EVs, they are seen as a step towards CO_2_ emission reduction and the green economy. However, they have a huge impact on the network. The main reason is that EVs are connected to the most critical part of the grid, the LV. This results in several difficulties for the SOs in addressing the increasing demand of energy, which is concentrated during the night hours when people return home and recharge their EVs [7,8,9].

Finally, but not less important, RES penetration is no longer an issue limited to the medium voltage (MV) portion of the DN. As a matter of fact, the high availability of RES solutions for small customers (such as house-sized photovoltaic panels and micro wind turbines) is increasing the complexity of the grid and of its management [10,11,12,13,14]. Furthermore, with the spreading of energy communities and microgrids, customers are becoming producers (the so-called prosumers), and they demand an active role among the grid players. Of course, on the one hand this is beneficial because all citizens may be aware of the network conditions and requirements. On the other hand, the modest transfer of power to the grid from RESs is critical from the SO’s perspective, who must adopt curtailment more and more frequently. Consequently, several algorithms addressing the better management of the grid and curtailment reduction can be found among the literature [15,16,17,18,19]. However, algorithms alone cannot solve the measurement requirements in such a harsh environment. ITs need to be capable of measuring voltages and currents in the actual status of the grid, whose levels of distortion can be more or less critical (but within the limits of the EN 50160 [20]). However, this condition is not always guaranteed, considering the evolving conditions of the grid and inadequacy of the relevant standards.

In light of the presented scenario, this paper aims to move toward the comprehension of the actual behavior of ITs during their operation in off-nominal, but realistic, conditions. In detail, a widespread sensor known as the Rogowski coil (RC) is tackled in this work. Its accuracy performance is not simply assessed when the measuring signal is distorted but when simultaneously different influence quantities (environmental and electrical) act on the measuring device.

In fact, it is noteworthy that several studies on RC behavior in the PQ frequency ranges (50 Hz to 2500 Hz) or even higher ranges can be found in the literature. For example, in [21,22] RCs are designed for measuring high-frequency components. The authors of [23] instead present an RC and a filtering stage to address the presence of distortion associated with the protective devices. RCs are adopted as the main sensing technology in [24] for measuring the supraharmonic content of distorted signals. Finally, in [25] a calibration system aimed at testing RCs at a high pulsed current is described.

However, to the authors knowledge there are no sufficient studies that focus on the actual operation of the RC considering several influence factors, in addition to the distorted signal, acting on the RC. To this purpose, an entire section in what follows is dedicated to the topic of influence quantities, aiming to clarify and highlight their importance.

Among the variety of influence quantities that may affect an RC, positioning, magnetic field (proximity), and burden are the ones studied in this paper. Therefore, using a dedicated measurement setup, developed by the authors of [26], three off-the-shelf RCs have been tested regarding burden, magnetic field, and positioning (one or more at the same time) while measuring the distorted currents. The results clarify the impact of actual measured conditions on the accuracy performance of the RCs under test.

The remainder of the paper is structured as follows. Section 2 provides a simple overview of the typical influence quantities acting on ITs and their role in the literature. A summary of the measurement setup developed in [26] is given in Section 3. The set of tests designed to assess the RCs is described in Section 4, while the measurement results and their associated comments are presented in Section 5. Finally, Section 6 provides the conclusion of the work with some suggestions for potential future works.

## 2. Influence Quantities

### 2.1. Introduction

The ideal IT’s operating conditions, achievable inside a research laboratory environment, are seldom found in the field. This is because ITs can be installed in a variety of either indoor or outdoor environments, in which several factor can singularly or simultaneously affect them. It consequently becomes crucial to assess, but first to find, the influence quantities that act on each type of IT in their peculiar location of installation.

According to the international vocabulary of metrology [27] an influence quantity is a “quantity that, in a direct measurement, does not affect the quantity that is actually measured, but affects the relation between the indication and the measurement result”. Therefore, the term influence quantity alone is not sufficient to describe all the potential operating conditions that may affect an IT. For example, a first classification of terms could be:

- Influence quantity, such as temperature, humidity, moisture, electromagnetic fields, pressure, altitude, burden, etc.
- Influence factor, such as the level of distortion of the measured signal (hence the quality of the source) compared to the rated, the design characteristics, etc.

All together they represent the parameters to fix and/or assess in order to gain a complete knowledge of the conditions under which ITs operate.

### 2.2. Literature

Considering the significance of such a topic, the literature already provides interesting works in which several influence factors and quantities are studied.

As one of the most influential quantities, temperature effect is commonly studied for all kinds of devices. For example, a new method for an online compensation of the temperature effect is presented in [28] for electronic ITs (EITs). The accuracy behavior over time of low-power ITs (LPITs) is studied in [29]. Finally, in [30] the metrological characterization of a calibration system aimed at testing inductive ITs over temperature is described. Of course, the above literature refers to air temperature affecting the ITs. However, various other papers can be found on different electric assets and different temperature evaluations (on components, oil, insulation, etc.), but these are beyond the scope of this paper.

Now we turn to another influence quantity, humidity, which is seldom considered in the literature as far as ITs are concerned. What can be found is mostly dedicated to the moisture content of power transformers’ insulating materials (e.g., oil), see [31,32].

Pressure is another quantity that is used to assess ITs’ health. For example, in [33] and [34] new techniques were developed to measure the oil pressure inside high-voltage (HV) voltage transformers (VTs).

A key role in performing measurements is assumed by the burden connected to ITs. In fact, it allows for adjusting the device output to a level suitable for data collection. Furthermore, the rated burden sets the IT in an optimal operating condition. To this purpose, the literature is very vivid and active. For example, [35,36] describe a calibration system and calibration methods, respectively, for burdens to be used for ITs.

For the sake of brevity, the last considered influence quantities are position and magnetic fields. Such quantities mainly affect current transformers (CTs) because (i) in case of window type CTs, the uncentered position with respect to the current carrying cable causes degradation of the CTs accuracy; and (ii) the position of the surrounding conductors is a source of magnetic fields that influence the measurements performed by the CTs. Consequently, in [37] a new EIT has been developed that minimizes the impact of positioning, while a new method to compensate it has been presented in [38].

### 2.3. Standards

After presenting the state-of-the-art studies available in the literature, the standards need to be analyzed as well. As far as ITs are concerned, the reference standard is the IEC 61,869 series. It contains several documents, including IEC 61869-1 [39] and -6 [40], which give the general requirements for inductive ITs and LPITs, respectively. Afterwards, the remaining documents tackle specific ITs. For example, IEC 61869-2 [41] and -3 [42] deal with inductive CTs and VTs, respectively, while IEC 61869-10 [43] and -11 [44] describe low-power CTs (LPCTs) and VTs (LPVTs), respectively. The series includes other documents, whose content is beyond the scope of this work.

As for the influence quantities or factors, in [39] the authors focus on (i) the air temperature categories in which ITs are classified (all ranges remain within −40 °C to +40 °C); (ii) the maximum altitude at which an IT can be placed; (iii) the air pressure; and (iv) the relative humidity operating range. Afterwards, the standard describes some type tests to be performed on the ITs, among which the influence quantities are included only in the windings temperature rise test and the electromagnetic compatibility (EMC) test. to the work of [41,42] do not introduce any other information on the topic with respect to [39].

However, some novelties are introduced in the LPITs documents of the standard. First, [40] specifies the rated burden for LPITs, which is a 2 MΩ, 50 pF impedance. Second, the EMC tests are much more detailed, and new tests are added compared to [39]. Third, a test to determine air temperature is defined. It specifies the cycle (with max and min temperatures) to be used for testing the LPITs. A small note on the rated burden: manufacturers seldom specify or even comply with the defined rated value.

Moving to [43], the description is even more detailed. The accuracy tests section includes a variety of new tests to assess LPCTs. For example, a test for determining frequency, a test for determining the accuracy and position of the primary conductor, and a test to assess the impact of the magnetic field produced by adjacent phases. As for the positioning tests, a dedicated annex is given in [43], and more details are presented in the following sections when the tests are described.

Finally, [44], only adds a specific test for assessing the electric field impact from other phases. Furthermore, considering the structure of a LPVT, the tests vs. positioning are meaningless, and hence, not treated in the standard.

All in all, what is missing from both the literature and the standards is the analysis of the Its’ behavior when they are simultaneously affected by several influence factors or quantities. Therefore, the added value of this work can be summarized in the accuracy performance assessment of RCs when subjected to multiple influence quantities and while measuring distorted signals. This might significantly contribute to the future versions of the relevant standards, such as the ones previously introduced.

## 3. Measurement Setup

This section briefly summarizes the measurement setup developed in [26] for testing RCs. It also describes the novelties introduced for the research performed in this work.

**Figure 1:**
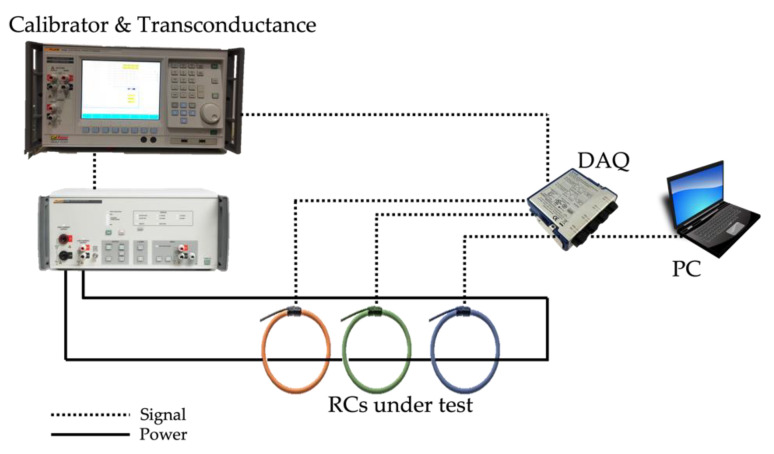
Schematic of the adopted measurement setup.

Figure 1 shows the measurement setup. It consisted of two main parts, the signal generation and the data acquisition. The former part was obtained with a calibrator in addition to its transconductance, a Fluke 6105A and a Fluke 52120A, respectively. They guaranteed a stable current up to 120 A with an accuracy of 0.009% of output, 0.002% of range, and <0.035° for the phase angle up to 850 Hz, and 0.04% of output, 0.004% of range, and 0.25° for the phase angle up to 3 kHz.

The acquisition stage instead consisted of a personal computer (PC) that acquired the data collected by a NI 9238 data acquisition board (DAQ). Its characteristics of interest are collected in Table 1.

The DAQ receives four signals: the three output voltages of the RCs under test (without any integrating stage) and a phase reference signal from the calibrator that is used for computing phase angle variations.

Such a measurement setup was used to test 3 off-the-shelf RCs produced by 3 different manufacturers. From here on they are referred to as R1, R2, and R3 for the sake of privacy. In fact, the purpose of this study was not to judge the manufacturer but to run objective tests with a focus on the RCs’ accuracy.

All RCs featured the same transformation ratio of 100 mV/1 kA and frequency ranges that include the PQ frequency range (50 Hz to 2500 Hz). R1 and R3 had an accuracy class (AC) of 1%, while that of R2 was 0.5%. More details on the RCs can be found in [26].

## 4. Experimental Tests

### 4.1. Overview

The measurement setup described in Section 3 was used in this work to assess the performance of 3 commercial RCs measuring distorted signals while affected by different influence quantities. Therefore, Section 4.2 describes the tests performed with different burdens connected to the RC. The position of the primary conductor with respect to the RC is tackled in Section 4.3. Finally, Section 4.4 illustrates the tests that simultaneously applied all the non-idealities previously described.

Note that:

- For each test the sampling window was 200 ms, and the sampling frequency was 50 kSa/s.
- For each test 100 repetitions were performed to calculate a significant mean value.
- For each set of tests, the injected current assumed three different values: (i) a pure 50 Hz signal (referred to as signal X); (i) a 50 Hz signal with a mix of harmonics, which resulted in a THD = 4.8% (referred to as signal Y); and (iii) a 50 Hz signal with a mix of harmonics, which resulted in a THD = 9.2% (referred to as signal Z).

For both the distorted signals, the mix of harmonics was designed according to the limits specified in [20]; hence, the included harmonics were all odd and up to the 25th. The limits in [20] hold for the voltage. However, considering both the lack of accurate standard limits for the voltage and typical power factor values, it can be assumed that those limits are reasonable even for the current.

- The amplitude of the primary current was 100 A rms for all performed tests.

- All the measurement results obtained from the tests were used to compute the ratio error ε and the phase displacement Δφ, which are defined as:
  (1) $$\varepsilon =\frac{K_{r}U_{s}-I_{p}}{I_{P}}\times 100,$$
  (2) $$\Delta \varphi =\varphi_{s}-\varphi_{p},$$
  where $U_{s}$ and $I_{p}$ are the rms of the 50 Hz component of the RC output voltage and the primary current to be measured (provided with the calibrator), respectively. $K_{r}$ is the rated transformation ratio of the RC under test. Finally, $\varphi_{s}$ and $\varphi_{p}$ are the phase angles of the 50 Hz component of the RC output voltage and the primary current to be measured, respectively. This procedure holds even for the distorted signals Y and Z, for which other analysis are performed, as described in what follows:

- During the results presentation the effect of proximity was included in the positioning. The reason is that the standards include this type of test among the positions in which to assess the device accuracy.
- To avoid any confusion with the values in % when subtracted among each other, the ratio error is presented in terms of its absolute value. Therefore, the results in the graphs should be multiplied by 100 to obtain the % notation.

In summary, the following tests, in which different influence quantities act on the RCs under test, are performed once each for the signals X, Y, and Z.

### 4.2. Tests vs. Burden

The first set of tests aimed at assessing the influence of the burden on the overall accuracy of the RCs. The idea behind these tests is that most of the commercial devices’ manufacturers neither state their compliance with the IEC 61869 nor specify the rated burden. Furthermore, it occasionally a device requires a rated burden far different from the 2 MΩ defined in [40]. The result of these diverse approaches, all incompliant with the standard, is an overall confusion among the users, which is an erroneous way to approach the measurements.

Therefore, starting from the rated value, 5 different burdens were selected for testing the RCs. Their values and names are collected in Table 2. As it can be seen, B_1_ and B_2_ represent a 10% variation from the rated value. This choice was made considering the typical commercial accuracies of resistors. B_3_ and B_4_ have greater rated resistance values than B_1_ and B_2_. The former’s resistance was 1 MΩ, similar to most of the acquisition system or measuring instruments, while the latter had significantly higher values compared to 2 MΩ. Overall, the set of chosen resistors was used to scan a wide variety of burden configurations.

The 5 burdens were tested with the signals X, Y, and Z, resulting in 15 tests in this first set.

### 4.3. Tests vs. Position

**Figure 2:**
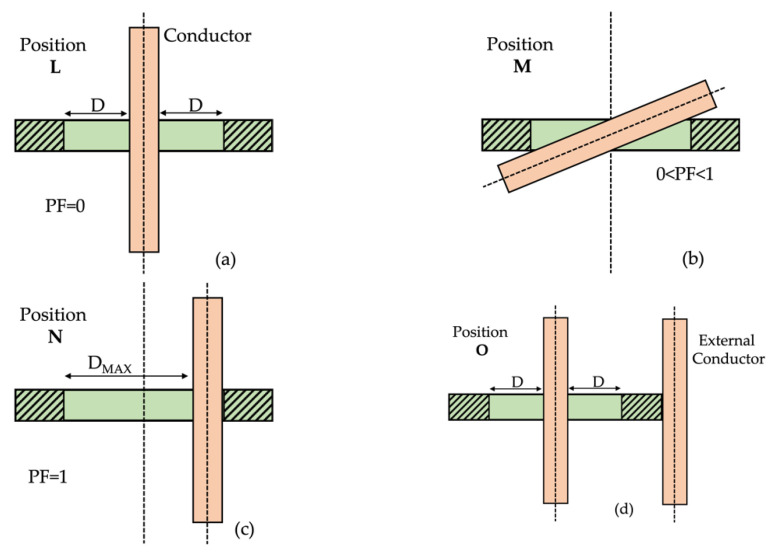
Four testing positions defined in [43] for RCs: (**a**) position L (**b**) position M (**c**) position N (**d**) position O.

The positions to be used for testing RCs are defined in [43] as described in Section 2.3. However, the standard prescribes testing each position with the ideal (50–60 Hz) signal. Figure 2 summarizes the 4 positions defined in [43]. The pictures illustrates the concept of position factor (PF), which varies between 0 and 1 and quantifies the position of the primary conductor (light brown) with respect to the RC (green). A PF = 0 corresponds to a perfectly centered primary conductor (referred to as position L). A PF = 1 instead reflects a primary conductor that is on one side of the RC but is still perpendicular to it (position N). In between the limits of PF, there is an intermediate position—not specified in the standard and up to the RC tester—which corresponds to a primary conductor with an angle of <90° with respect to the RC axe (referred to as position M). Finally, position O is the same as position L but with an external conductor close to the outer surface of the RC. This last position aimed at testing the effect of external magnetic fields generated by other phases or return conductors.

In summary, the four positions L, M, N, and O were tested with the three signals X, Y, and Z, resulting in 12 tests for this second set.

### 4.4. Tests vs. Burden and Position

Section 4.2 and Section 4.3 present tests in which position and burden act singularly on the RCs under test, measuring either ideal or distorted currents. This last set of tests instead aimed to combine all the influence quantities and factors together. This way it is possible to assess the RCs’ behavior and the effects on their accuracy.

Therefore, combining 3 signals, 4 positions, and 5 burdens, the result was an overall of 60 tests.

## 5. Experimental Results

For the sake of clarity, this section is structured as Section 4. Therefore, Section 5.1 presents the results of the tests to determine burden, while Section 5.2 contains the results of the tests for position. Finally, Section 5.3 describes the results of the combined tests, as introduced in Section 4.4. Note that precise comments are included in each section immediately after the test results.

### 5.1. Tests vs. Burden Results

Once fixed and installed, it is rarely possible to change the burden of an IT. Therefore, it is a good practice to calculate the correct value of the burden and then characterize it before the installation. This is performed to prevent any incorrect behavior of the IT, even at the rated condition of the signal to be measured.

#### 5.1.1. Signal X

**Figure 3:**
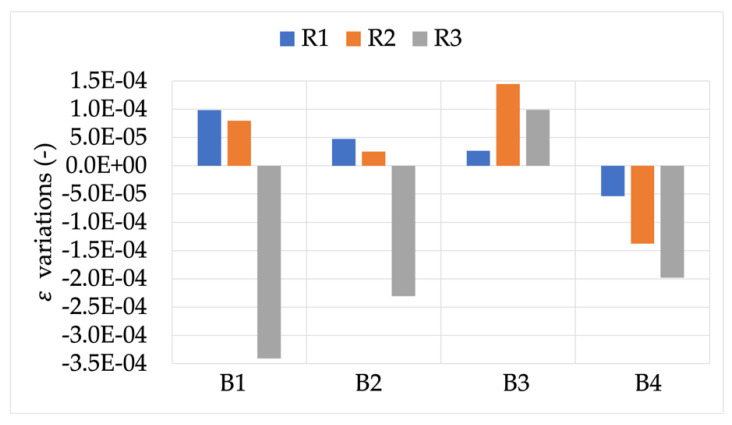
ε variations, when measuring signal X, for burdens B1 to B4.

**Figure 4:**
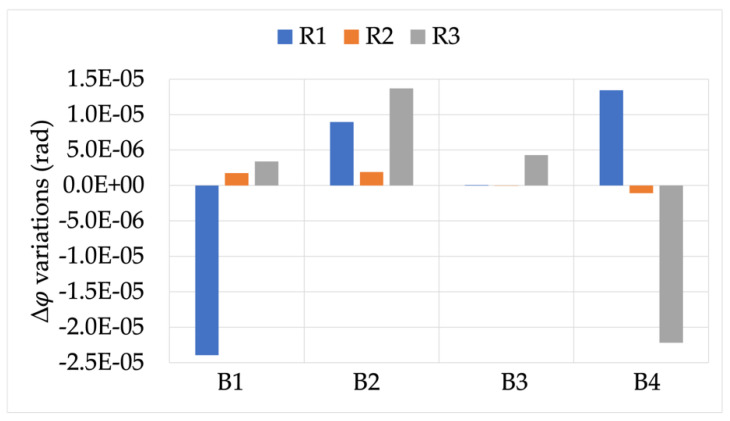
Δφ variations, when measuring signal X, for burdens B1 to B4.

The first result then was the effect of burden on the accuracy of the RCs under test measuring signal X (the rated one). Figure 3 and Figure 4 depict the ε and Δφ variations, respectively, compared to the rated burden B_R_. Starting with these two figures, a color code is used to distinguish R1, R2, and R3 (blue, orange, and gray, respectively).

Looking at Figure 3, an overall comment is that each RC acts differently depending on the burden value. Furthermore, none of them experience variations comparable to their accuracy class’s limits (1% and 0.5%) but always at least one order of magnitude lower. Specific comments, instead, arise from the graph. In particular, R1 was almost insensitive to all burden values; R2 was mostly affected by the 1 MΩ resistor, while R3 reacted with the highest variation for all burdens (up to a 3.5 $\times 10^{-4}$).

Almost the same comments stated for ε can be extended to Δφ. In fact, the highest variations were in the order of −2.5 $\times 10^{-5}$. Furthermore, while R1 and R3 showed visible variations vs. burden, R2 appeared completely insensitive to the different burdens.

A final comment from this first assessment is on the measurements’ accuracy. For both ε and Δφ the standard deviation of the mean was computed (from the repeated measurements), obtaining maximum values in the order of $10^{-7}$ and $10^{-8}$ for ε and Δφ, respectively (worst case among the three RCs).

#### 5.1.2. Signals Y and Z

**Figure 5:**
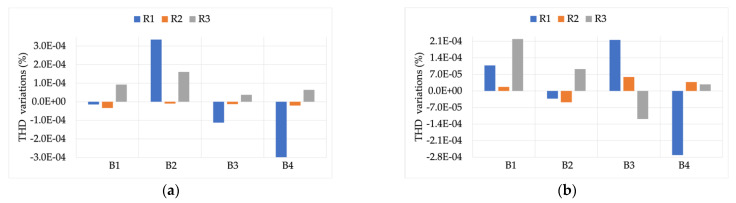
THD variations, compared to the reference values, vs. burden. (**a**) RCs’ measuring signal Y with THD 4.8%; (**b**) RCs’ measuring signal Z with THD 9.2%.

**Figure 6:**
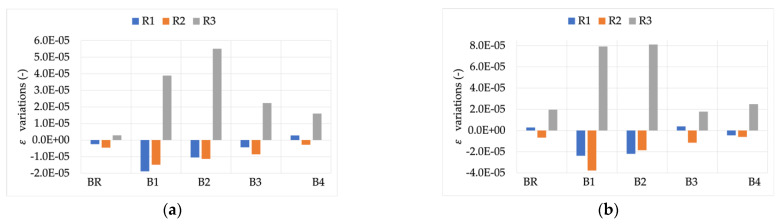
ε variations, compared to the reference values, vs. burden. (**a**) RCs’ measuring signal Y with THD 4.8%; (**b**) RCs’ measuring signal Z with THD 9.2%.

**Figure 7:**
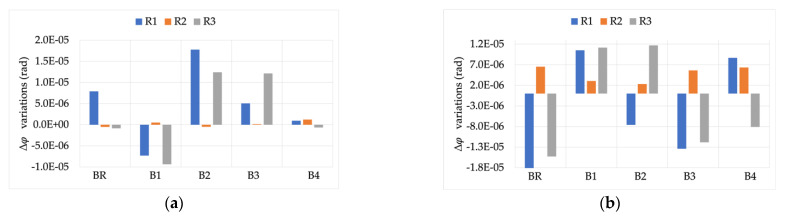
Δφ variations, compared to the reference values, vs. burden. (**a**) RCs’ measuring signal Y with THD 4.8%; (**b**) RCs’ measuring signal Z with THD 9.2%.

At this point the focus of the results analysis can be moved to the tests performed with the distorted signals Y and Z. To this purpose, Figure 5, Figure 6 and Figure 7 are used.

Figure 5 shows the effect of burden variations (B1 to B4) on the measurement and computation of THD. The graph (a) on the left represents the case in which the RCs are measuring the signal Y with a THD of 4.8%, while the graph (b) on the right represents the case in which the RCs are measuring the signal Z with a THD of 9.2%.

Both graphs in Figure 5 leave no doubts about the fact that a burden variation does not result in a degradation of the measurement capabilities of the RCs while measuring distorted signals. In detail, the maximum variation observed was −2.8 $\times 10^{-4}$ for R1.

In support of this, Figure 6 and Figure 7 show the difference between the accuracy parameters (ε and Δφ) computed for signals Y and Z and those computed for signal X. In other words, the aim was to assess whether the measurement of the module and phase of the 50 Hz components is affected by the presence of harmonics. Note that, as detailed in Section 4.1, ε and Δφ were always calculated for the 50 Hz component.

The results in Figure 6 lead to two conclusions: (i) R3 is highly sensitive to the presence of distortion inside the current to be measured. The measured variations, with respect to the rated signal, were twice those measured for R1 and R2; and (ii) for all RCs there was a slight dependency on the THD value. In fact, in all cases and burdens, the variations measured for signal Y were lower than those measured for signal Z.

The same comments cannot be extended to the phase angle evaluation. In fact, there was no dependency on the THD value, and all the variations were quite modest and in the order of few or tens of microradian.

In light of what presented in Section 5.1.1 and Section 5.1.2 it can be summarized that (i) with the exception of R3, the effect of the burden variation has no significant effect, compared to the accuracy limits of the RCs, on the accuracy of the RCs while ideal or distorted signals are being measured; and (ii) Δφ, compared to ε, is much more immune to the effects of the burden on the presence of harmonics inside the signal to be measured by the RC. However, even if limited, the burden has some effects on R3; therefore, it is worthy to stress the need of tests vs. burden or to force manufacturers to adopt a standardized burden.

### 5.2. Tests vs. Position Results

In this section the effect of the RC position on its accuracy while measuring distorted signal is analyzed. Note that the assessment of the single effect of position is not the focus of this paper because it has been already stressed in [45]. Furthermore, all results are compared to the centered position L and performed at the rated burden B_R_.

#### 5.2.1. Position M

Position M is the one with a PF between 0 and 1 (see Figure 2) and, like position N, it occurs when the RCs are not fixed and centered on the cable at the installation point.

**Figure 8:**
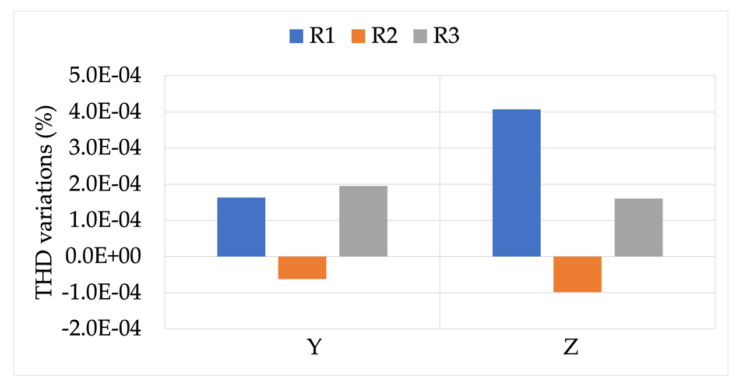
THD variations, compared to position L, obtained from measurements at the position M.

The first analyzed result is graphed in Figure 8. It contains the variations of the THD computations, obtained while measuring signals Y and Z, compared to the same signals measured for position L. From the graph it can be observed that, on the one hand the absolute value of the variation was quite limited for both signals Y and Z. On the other hand, R1 and R2 showed a little dependency on the THD value, while the opposite can be stated for R3. Therefore, the THD evaluation was not affected by position M.

**Figure 9:**
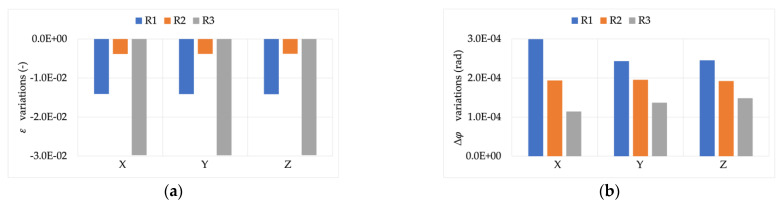
ε and Δφ variations ((**a**,**b**), respectively), compared to position L, for the three signals measured with position M.

The module and phase of the 50 Hz component were also evaluated. To this purpose, Figure 9 presents ε and Δφ variations (left and right graphs, respectively) computed while measuring the three signals, compared to what was obtained for position L.

From graph (a) it is clear how the effect of THD is neglected by the one due to position (two orders of magnitude higher compared to what observed in Figure 8). In fact, the behavior of ε variation was almost constant for the three signals. From graph (b), instead, note that R1 and R2 Δφ variations were not affected by the simultaneous presence of position M, and a distorted signal was measured. In fact, the significant variation of such a graph can be attributed only to the position, as described in [45]. However, R3 differently reacts to the combined presence of the two non-idealities. In fact, the higher the THD, the higher the measured Δφ variation. This is another example of how combined influence quantities or factors affect the accuracy of a single device.

#### 5.2.2. Position N

**Figure 10:**
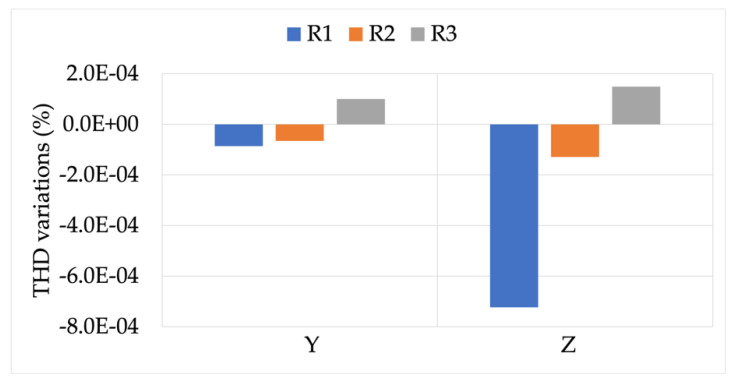
THD variations, compared to position L, obtained from measurements at the position N.

**Figure 11:**
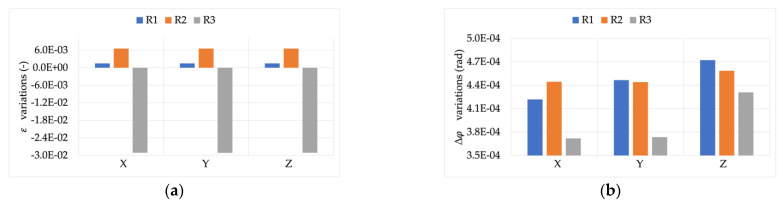
ε and Δφ variations ((**a**,**b**), respectively), compared to position L, for the three signals measured with position N.

Position N is characterized by the primary conductor being perpendicular to the RC axe but attached to one of its sides (see Figure 2). Analogously to what presented for position M, THD, ε, and Δφ variations are graphed and included in Figure 10 and Figure 11, respectively.

Starting from Figure 10, note that what was observed in Figure 9 can be confirmed even for position N. In fact, the absolute variation of THD is always very limited and almost negligible. In addition, the R1 dependency on the THD value can be confirmed and is quite evident from the graph.

Turning to Figure 11, graph (a) depicts ε variations that are coherent with Figure 9a, in which the THD effect is neglected by the one due to the position. However, another confirmation comes from graph (b), in which R3 shows again a dependency on the THD value as far as Δφ variations are concerned. Furthermore, even if limited, R1 and R2 also present a Δφ variation changing with the THD value. This effect can be attributed to the severity of position N compared to the previous one.

#### 5.2.3. Position O

Position O is the fourth position considered in [43]. It is also the position that assesses the effects of the external magnetic field acting on the RC under test. Therefore, a dedicated work was undertaken ([26]), and just a brief recall of the results is summarized here.

**Figure 12:**
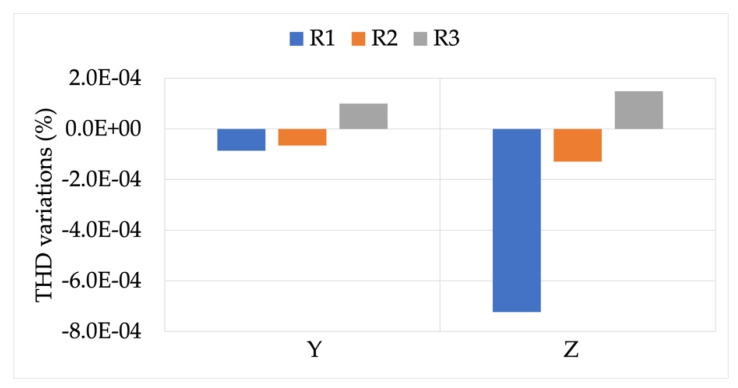
THD variations, compared to position L, obtained from measurements at the position O.

**Figure 13:**
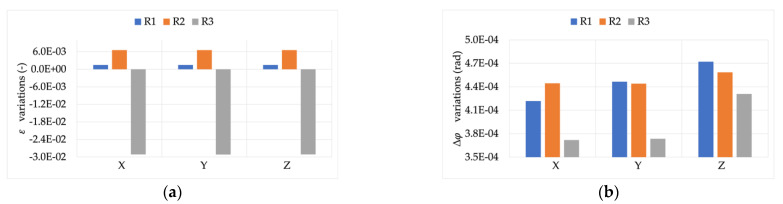
ε and Δφ variations ((**a**,**b**), respectively), compared to position L, for the three signals measured with position O.

Figure 12 shows the THD variation while measuring signals Y and X at the position O. Note how the results are fully aligned with what was obtained with both positions M and N. The same comments can be extended to Figure 13 for both graphs (a) and (b), which contain ε and Δφ variations, respectively (always for the 50 Hz component).

Overall, after evaluating the effect of non-centered positions of the primary conductor, with respect to the RC, while measuring distorted currents it can be concluded that (i) the effect of the distortion is negligible on the absolute value compared to the effect of position when acting singularly; and (ii) the combination of the distortion and the position does not always result in the superposition principle. In fact, it has been observed that for R3 the combination of distortion and position results in a higher degradation of the RC accuracy.

### 5.3. Tests vs. Burden and Position Results

This section contains the results of the tests with higher complexity in terms of influence quantities and factors affecting the RC measurements. However, it is also the most accurate test considering, for example, typical in-field conditions inside a secondary substation or inside an electric cabinet. Consequently, for the sake of reader comprehension, the following graphs have been prepared as coherently as possible along with those in Section 5.1 and Section 5.2.

**Figure 14:**
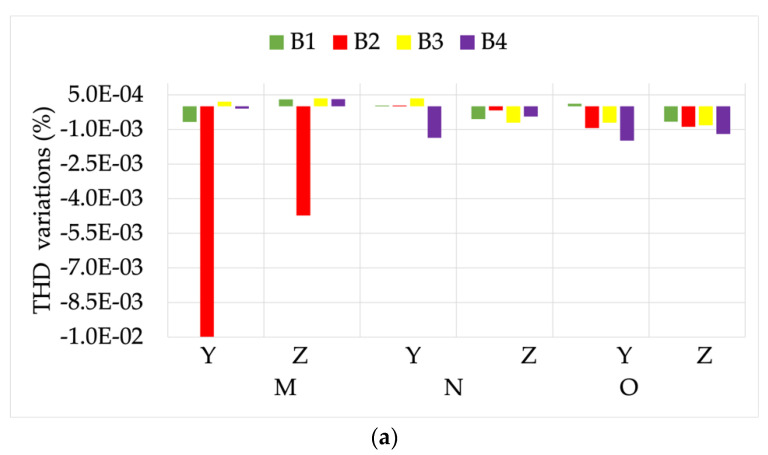

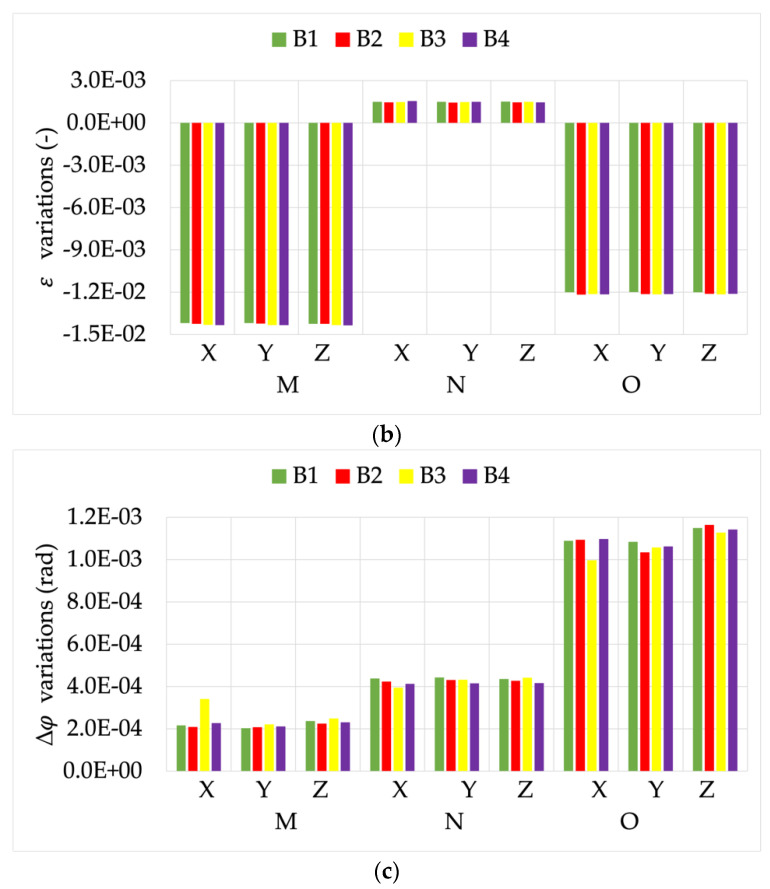
(**a**) THD, (**b**) ε, and (**c**) Δφ variations, from the tests vs. burden and position, for RC R1.

Let us start by describing Figure 14. From top to bottom it contains three graphs: (a) THD variations, (b) ε variations, and (c) Δφ variations for R1. All variations compare the measurements performed with rated burden and centered position L with those performed when influence quantities and factors were acting on the RC. Therefore, in each graph can be distinguished by the three non-centered positions M, N, and O; the three signals X, Y, and Z; and the four burdens B1, B2, B3, and B4. For the sake of clarity, a color has been assigned to each burden: green, red, yellow, and purple for B1 to B4, respectively. The same structure is used also for R2 and R2 results in what follows.

In graph (a), the almost negligible effect of all the influences on the THD evaluation should firstly be pointed out. The only exception was observed for the combination of B2 with the positions M and N. However, the absolute value of this effect was limited compared to the THD value computed.

As for the general trend, note from graphs (b) and (c) how the different positions affected ε and Δφ differently. In fact, positions M and O were critical for the ratio error, while only position O and signal Z had a strong impact on the phase displacement.

In graphs (b) and (c), note how many comments given in the previous sections can be confirmed. For example, the burden variation did not affect the accuracy of R1 in any of the tested positions, with the exception of some slight variations (see (c) combinations M–X and O–Z).

**Figure 15:**
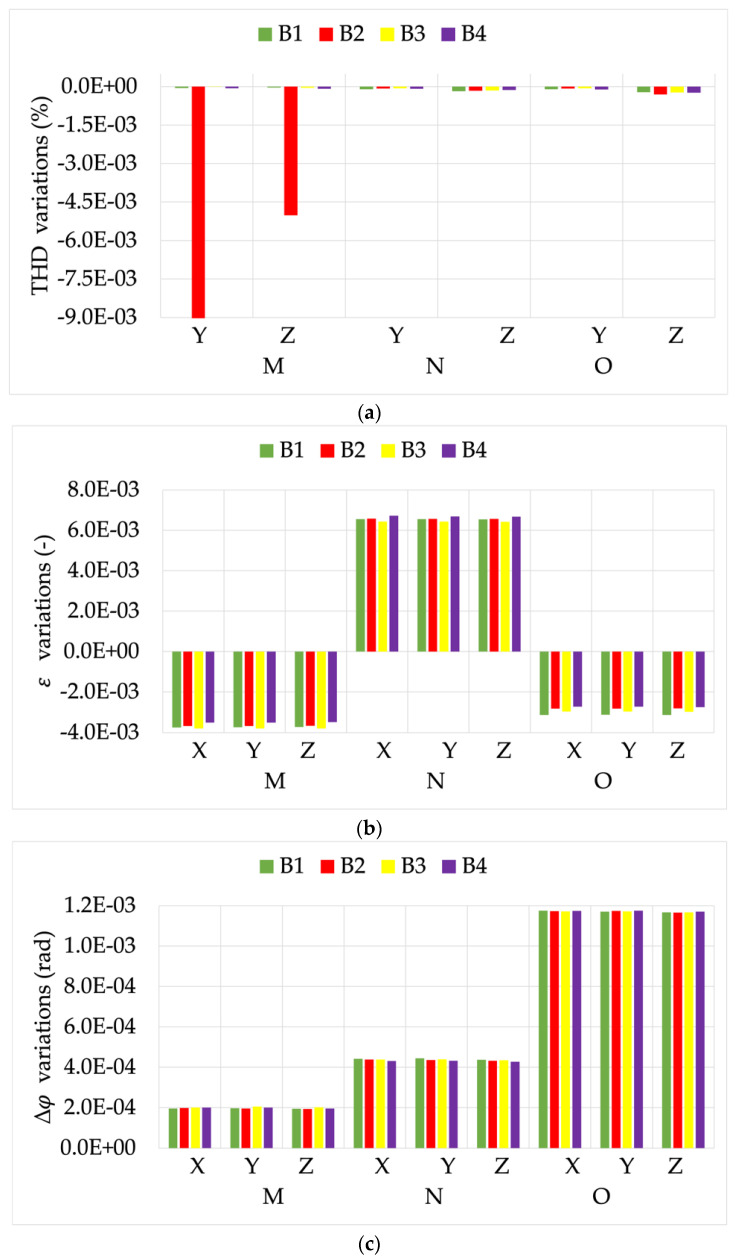
(**a**) THD, (**b**) ε, and (**c**) Δφ variations, from the tests vs. burden and position, for RC R2.

Analogously to what was presented for R1, Figure 15 shows the same results for R2. Comparing the results with those in Figure 14, (i) burden B2 was again critical for position M; (ii) position O, hence the presence of external magnetic fields, also affected the phase of R2; (iii) conversely to R1, ε in the case of R2 was sensitive to position N. This confirms that each RC has peculiar manufacturing solutions. Hence, when exposed to various influence quantities they respond in different ways.

**Figure 16:**
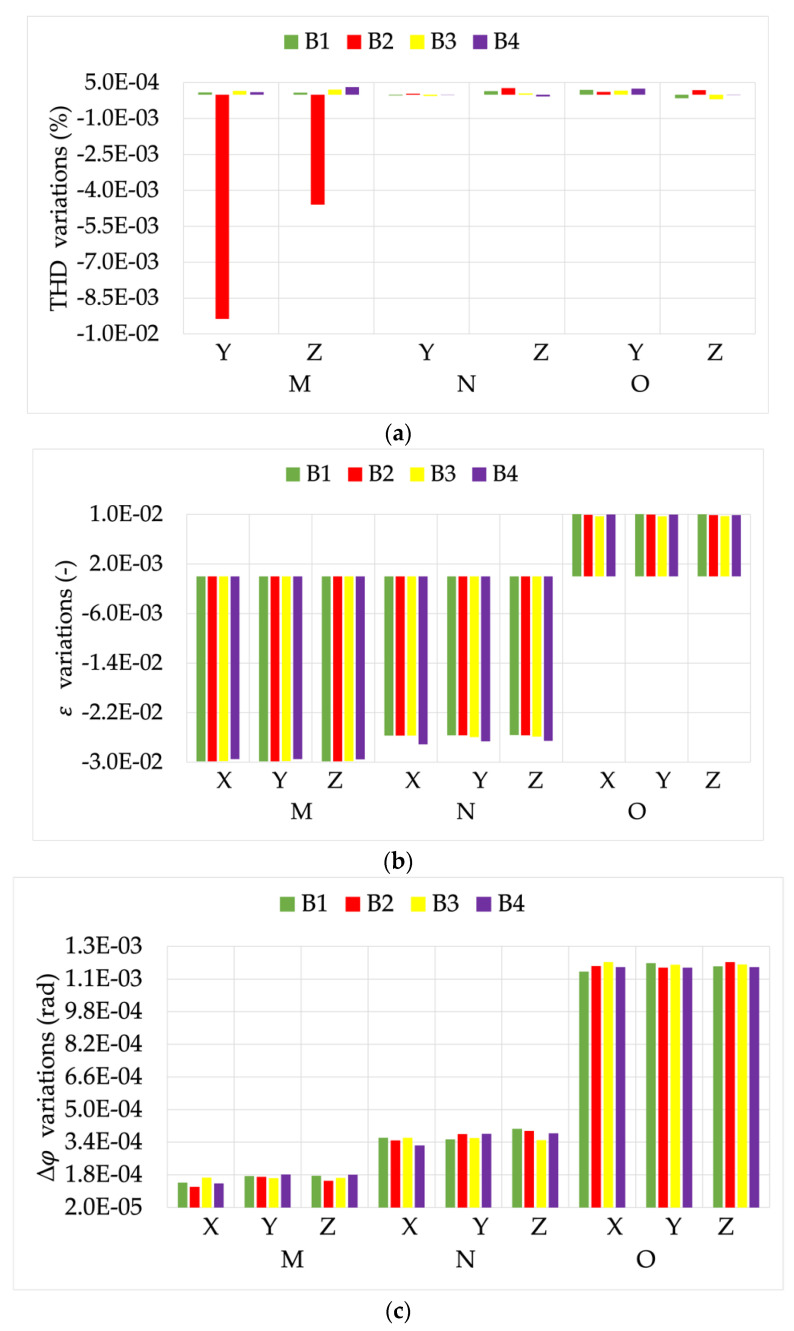
(**a**) THD, (**b**) ε, and (**c**) Δφ variations, from the tests vs. burden and position, for RC R3.

Finally, Figure 16 depicts the results from the tests on R3. It can be noted from graphs (b) and (c) that there was a slight dependency on the burden for some of the positions. This confirms that the combination of two or more non-ideal conditions may result in some unexpected behaviors.

Focusing on the phase presented in graph (c), note how the phase was again affected by the external magnetic field presence. ε, was highly sensitive to positions M and N.

All the comments on THD can be extended from the previous RCs results for R3.

Final overall comments are the following:

- There is no unique design and manufacturing technique to produce RCs. Therefore, one cannot assume similar behavior among RCs, either for ideal or distorted conditions.
- The results obtained in the existing literature are confirmed from the new set of results, highlighting the already known criticalities in terms of accuracy.
- The effect of burden variations on RCs’ accuracy is very limited when it is evaluated alone and in presence of distorted signals. Therefore, the standard should consider loosening the requirements for RC burden.

- The presence of distortion, hence a THD ≠ 0, is not significant in most of the case for the evaluation of RC accuracy.

- The combined presence of non-rated burdens, distorted signals, and position diverse from the centered one results in behavior that is not shared among all RCs. Hence, it was worthwhile to test them to better understand the accuracy feature of the device.

It is fundamental then to alert manufacturers and users to the unavoidable presence of multiple influence quantities and factors acting on the in-field installed devices. In fact, it is implausible to consider the laboratory conditions as standard and replicable outside of these circumstances.

Consequently, future versions of the standards should include the consideration of significantly more influence qualities; hence they are worthy of dedicated testing.

## 6. Conclusions

This paper presented a study focused on the accuracy of Rogowski coils. In particular, the accuracy was analyzed when influence quantities and factors acted on the devices. No comparison with other technologies was given, considering the specific focus on the RC behavior. The influence quantities considered included the burden and the position of the device, while the measured influence factor was the distortion inside the current. A specific setup, previously developed and described, was used to test three commercial Rogowski coils from different manufacturers. The results clearly emphasize the need for specific tests to be developed for each of the influence quantities and factors. Of course, not all of them have the same effect on the Rogowski coils, but their combination may be critical in some cases. In particular, it was found that the burden has a limited effect on the accuracy, while positioning has the greatest impact. Furthermore, the combination of influence quantities results in different behaviors among the RCs. This supports the need for testing to assess the accuracy of the devices.

Future works will take further steps in the direction forged by this work. More influence quantities and factors will be considered either for Rogowski coils or other medium voltage sensors.

## Figures and Tables

**Table 1:** Main characteristics of the NI9238.

Converter	24-bit	Voltage Range	±500 mV
Sampling Frequency	50 kSa/s/Ch	Input Impedance	>1 GΩ
Simultaneous Channels	Yes	Gain Error	±0.07%
Offset Error	± 0.005%	Input Noise	3.9 μ V

**Table 2:** List of burdens selected for the tests.

Name	Value (MΩ)
B_R_	2
B_1_	1.8
B_2_	2.2
B_3_	1
B_4_	1000
